# Adopting e-government to monitor public infrastructure projects execution in Nigeria: The public perspective

**DOI:** 10.1016/j.heliyon.2023.e18552

**Published:** 2023-07-23

**Authors:** Peace Afieroho, Robert Perkins, Xiyu (Thomas) Zhou, Bogdan Hoanca, Greg Protasel

**Affiliations:** a College of Business and Security Management, University of Alaska Fairbanks, AK 99775 USA; b College of Engineering and Mines, Univeristy of Alaska, Fairbanks, AK, 99775, USA; c College of Business and Public Policy, University of Alaska, Anchorage, AK, 99508, USA

**Keywords:** Public infrastructure project, E-Government, Public participation, Unified theory of acceptance and use of technology, Nigeria

## Abstract

Public infrastructure projects (PIPs) are critical to the socioeconomic development of any country and similar to most public activities, their governance requires effective public participation to be successful. Information and communication technology adoption in government-public engagements (i.e., e-government) has improved public participation in governance in developed countries. This study utilizes the unified theory of acceptance and use of technology to investigate determining factors for the Nigerian public to adopt e-government tools to promote public participation in monitoring PIPs execution. It adopts questionnaire survey and structural equation modelling techniques to show that performance expectancy, facilitating conditions, social influence, and effort expectancy significantly and positively affect behavioral intention to adopt e-government tools to monitor PIPs execution in Nigeria. This study provides scholars with an exploratory baseline for extension of e-government adoption to public infrastructure project management. This study also provides recommendations to policy makers, government technocrats, and project engineers on the need for policy changes, creation of interactive and up-to-date project websites for PIPs in Nigeria.

## 1 Introduction

According to the World Economic Forum [1], “infrastructure undergirds commercial life, provides vital social services, and supports human interaction round the world and across the street,” making it essential for economic development and critical to quality of life in a society. The importance of good infrastructure to sustenance and improvement of quality of life demands that nations must continue to invest in infrastructure development. The delays to infrastructure projects in the last decade, given the recent global economic recession and inability of infrastructure development to keep pace with demographic and political changes, has increased the urgency for these investments. To close the global infrastructural deficit, an annual investment of about $2.6 trillion will be required till 2030 [2]. The recent $1.2 trillion bill for infrastructure development passed by the US legislature to close infrastructure development gaps emphasizes this urgency [3].

Public infrastructure projects (PIPs) have a low success record in developed and developing nations, given their poor delivery. In Nigeria, most PIPs fail or are as ineffective as most government services [4,5]. Generally, while most government service programs are known for being ineffective, government-public engagement in documentation and transactional services (e.g., getting birth certificates and driving licenses) has seen improvements in most developed nations. Such improvements stem from augmenting service delivery with information communication technology (ICT) platforms. ICT is a good enabler of public participation and empowerment, encouraging greater transparency and better public service delivery [6]. It enhances the methods of delivery of government services and the public's access to those services, via use of technology.

E-government entails integration of ICT into government processes to enhance efficiency. The purpose is to enable the public and businesses to better access government services via diversified communication channels. E-government adoption is admired for its promise to solve socioeconomic problems and improve a country's capacity to address public needs and demands, especially in developed countries with mature ICT infrastructure [7,8].

ICT tools (e.g., the Internet, websites, and mobile phones) are prevalent in Nigeria, despite its developing nation status. Thus, the Nigerian government can leverage existing ICT tools to adopt e-government effectively and efficiently to enhance government-public engagement in delivering government objectives, including infrastructure development projects. Using e-government tools and platforms, information can be shared, displayed, and discussed among public with ease [6]. For example, effective public discussion of progress of a PIP can help discourage corruption and deviations from project objectives during implementation, thereby helping to improve its chances of successful completion. However, there has been little deployment of e-government to enhance public participation in monitoring PIPs execution in Nigeria. E-government deployment in Nigeria is currently low, with the country ranking 140th out of 193 countries in the 2022 UN e-government maturity survey [9]. E-government has been used for only basic government services, such as citizen passport, university entrance examinations, etc., in Nigeria.

There are several behavioral studies on adoption of ICT tools to personal commerce and delivery of basic government services [[10], [11], [12], [13], [14], [15], [16], [17]]. Generally, these studies held adoption of ICT tools to be a positive enabler to effective client-customer engagement in personal commerce or basic government service delivery. However, few studies probed e-government adoption to improve government-public engagement [18,19] in governance, and to the best of our knowledge, there is none with particular focus on public perspective to public participation in monitoring PIPs execution in Nigeria (see Table 1). To bridge this gap in literature, this study addresses the following research question: What are the determining factors for public willingness or behavioral intention to adopt e-government tools and approaches to participate effectively in monitoring PIPs execution in Nigeria?

**Table 1:** A cross-section of some UTAUT model studies and their application areas.

	Energy efficiency technologies	Online shopping and banking	Public services including public health and universities	Public-government engagement	Public infrastructure project management
Application of UTAUT	[20,[21], [22], [23]]	[13,[24], [25], [26]]	[14,17,27,[28], [29], [30], [31], [32]]	[[10], [11], [12],^18^,^19^]	None
Countries	Iran, Latvia, Japan, Australia	Nigeria, Serbia, Kuwait, Tanzania	Malaysia, Nigeria, Bangladesh, USA, UK Kenya, Pakistan, Zambia.	Portugal, Slovenia, Indonesia, Jordan	None

This study takes the novel approach of extending and adapting technology adoption models from personal commerce and basic government service delivery to government-public engagement in public infrastructure project management. It extends the frontier of studies in e-government adoption in Nigeria by presenting new empirical results and extending application of e-government to government-public engagement, a different aspect of e-government application, which to the best of our knowledge, has not been studied before in Nigeria.

This study also identifies factors that facilitate e-government adoption to incentivize effective public participation in monitoring PIPs execution in Nigeria, from the public perspective. Effective public participation can enhance accountability and the successful delivery of PIPs, enhancing the quality of life. The findings from this study provide valuable insights for public policy practitioners, government technocrats, and project engineers on how to facilitate effective public participation to support successful delivery of PIPs to enhance the standard of living in society.

The rest of this paper is structured in sections. Section 2 is a literature review of current studies on e-government and its adoption in Nigeria, supporting theories of e-government and their extension to PIP management, identification of gaps in current literature and proposed hypotheses to explain the gaps. Section 3 presents the research methods which describe the questionnaire survey and statistical modelling techniques used for data analysis. Section 4 presents the survey results and testing of hypotheses and Section 5 discusses the results, conclusion, and recommendations along with the study limitations.

## 2 Literature review

### 2.1 E-government and its adoption in Nigeria

E-government can be broadly described as ICT deployment to achieve a more efficient and effective government by leveraging it for vigorous and effective public participation in governance. The World Bank [33] notes that technology adoption for e-government serves various objectives, including “better delivery of government services to citizens, improved interactions with business and industry, citizen empowerment through access to information, or more efficient government management.” E-government focuses on using ICT to encourage more purposeful public participation in government via easy access to information and government officials, thereby inducing more effective government-public engagement and good governance.

In recent times, most public administration and ICT experts have projected e-government as a solution to government inefficiencies and corruption. It is also presented as a good governance, transparency, and accountability enabler [33,34]. In most developed nations, the government employs ICT to interact with the public, promote transparency, and involve stakeholders in development of public policies and societal development**.** However, in developing nations such as Nigeria, government-public interaction is poor; even the few instances where ICT is leveraged seems tokenistic. Similar to most developing countries, Nigeria faces the need for more government-public interaction to facilitate good governance and development of basic PIPs (e.g., markets, schoolhouses, and roads), which should be provided by good governments [5,34] for public benefits.

Nigeria is a significant developing nation, with the largest population, and among the most dynamic economies in Africa. It consists of six geopolitical regions and has been under democratic civilian rule for over 20 years. Even then, societal and economic development in Nigeria is poor, with a low GDP per capita of $2,300, and poor government service and infrastructure delivery. Further, government-public engagement is poor, as the public cannot hold government officials accountable [5,35,36]. This situation has contributed to corruption and poor achievement of infrastructural development goals. Thus, the Nigerian public do not trust the government to effectively drive infrastructure development. Hence, the private sector and entrepreneurs are stepping up to drive infrastructure development for business survival. A good example is the success story of telecommunications infrastructure development in Nigeria, driven by the private sector. Government procurement and PIPs development planning are usually shrouded in secrecy and have become the main circumstantial procedural vehicle used for funds diversion, misappropriation, and embezzlement. Currently, most basic government services in Nigeria rely on manual transactions and delivery. In recent times, however, there has been a shift to using e-government tools and platforms to deliver some public services (e.g., university entrance examination [e-jamb] and citizen passport [e-passport]) in Nigeria. This development has encouraged exploratory interest in e-government adoption to monitor PIPs execution in Nigeria.

Nigeria's efforts to adopt e-government began in the 2000s and it attained a high teledensity of 108% in 2022, though mostly driven by the private sector. Availability of ICT infrastructure and public willingness to adopt e-government service platforms are key requisites for widespread e-government utilization in a country. Several empirical studies examine the provision of ICT infrastructure to support implementation of e-government for public service delivery [[10], [11], [12],^19^,^27^,^37^]. These previous studies conclude that ICT infrastructure development and support by the government is required for effective adoption of e-government in a country. However, to the best of our knowledge, none of the studies investigated factors that will incentivize the public to be willing to adopt e-government tools to improve public engagement in monitoring PIPs execution, especially in Nigeria. Given the relevant Nigerian context, it is vital to focus on the Nigerian public to understand the factors that influence their behavioral intention to effectively adopt e-government tools in monitoring execution of PIPs.

### 2.2 Theoretical models and hypotheses

Leveraging ICT for government-public is essential to e-government development. However, the availability and provision of technology infrastructure and platforms alone may not confirm and support effective e-government adoption by the public [27]. E-government's success depends on both government support and public willingness to accept, use, and adopt e-government service platforms. Empirical studies by Refs. [19,27,37] observed a low-level of maturity and public adoption of e-government services in Malaysia, even with significant government support for e-government. Therefore, public willingness to adopt e-government tools is also vital to success of e-government initiatives in a country. Hence, a good understanding of public willingness to adopt e-government tools to monitor PIPs execution in Nigeria, which is the aim of this study, is important.

**Fig. 1:**
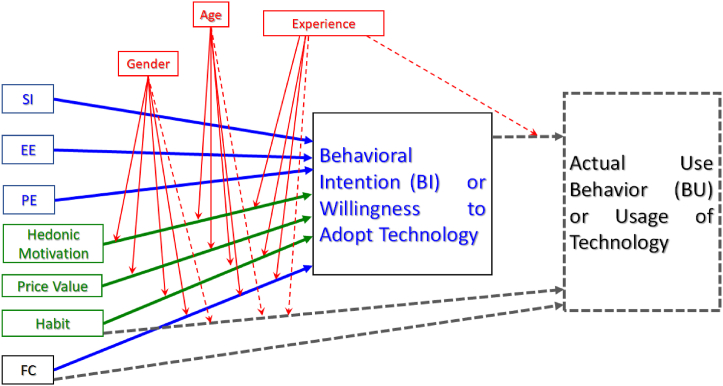
Extended unified theory of acceptance and use of technology (UTAUT); PE—performance expectancy, EE—effort expectancy, SI—social influence, FC—facilitating conditions.

Public acceptance or rejection of a new idea or system is closely linked to how well the public feels persuaded to modify their behavior towards the new idea or system [20]. Public adoption of a technology as a way of life can be viewed as a public behavior modification towards a new system and may be explained or predicted by behavioral theories and conceptual frameworks. Accordingly, several behavioral theories and conceptual frameworks have been proposed to explain and predict human behavioral intention to adopt a new technology or system. For example, the theory of reasoned action (TRA) holds that behavior can be explained and predicted based on attitudes, norms, and beliefs, but it is criticized for not recognizing the power of individuals over their behavior [38,39]. In an attempt to address some of the weaknesses in TRA, the theory of planned behavior (TPB) later introduced perceived control over behavior as an additional factor to explain and predict human behavior. TPB has been used to explain human behavioral intention to adopt energy efficiency technologies [[40], [41], [42]]. According to Ref. [20], TPB explains that intention is a key direct psychological determinant of behavior and is affected by attitude, subjective norms, and perceived behavioral control. With focus on a technology adoption behavior situation, another human behavioral intention model, technology acceptance model (TAM) was developed as an extension of TRA. It identifies perceived ease of use and usefulness of the technology [[20], [27], [37]] to be determinant factors of behavioral intention to adopt a technology but does not reflect the varieties of user's constraint [43]. However, all the aforementioned and previous models have been determined to rationally explain only about 40% of variations in technology adoption behaviors [27]. Venkatesh et al. [44,45] integrated several determinants from previous behavioral theories and technology use and adoption models such as TAM, TRA, TPB, etc., into one framework of four core determinants of behavioral intention to use technology which they described as the unified theory of acceptance and use of technology (UTAUT). UTAUT framework is one of the most popular frameworks for technology adoption models and has been validated to explain technology adoption behavior better than other models [27,[44], [45], [46]]. There are several variant models within the UTAUT frameworks, but the four core determinant factors in the framework are performance expectancy (PE), effort expectancy (EE), social influence (SI), and facilitating conditions (FC). UTAUT framework integrated determinant factors from other behavioral change and technology adoption models, for example, PE is closely related to usefulness of technology and EE related to ease of use of technology [43,46] in TAM. In variations, it also represents other contextual factors such as age, gender, price value, etc., as additional potential moderating or direct determinant factors (Fig. 1).

UTAUT has been modified and adapted in multiple ways to include different combinations of contextual factors. For example, price value and perceived safety gained from use of technology were added as contextual factor to UTAUT framework for adoption of technology to manage bicycle sharing system in Iran [20], hedonic motivation and perceived price value were additional contextual factors in solar power technology adoption studies in India [47], and technology awareness and culture influence were added as additional factors towards adoption of smart city technology for elderly people in Saudi Arabia [48]. Empowerment, social capital, trust, insecurity, gamification amongst other contextual factors were specifically included in the application of UTAUT models to explain technology adoption to participatory budgeting in Portugal [10,11]; electronic voting in local elections in Slovenia and Jordan [12,19], e-participation in Indonesia [18] etc. Few other authors [37,49] used the model to study e-government service adoption in Kuwait and Jordan, respectively, validating the UTAUT model. While the results vary, collectively from the aforementioned studies, PE, EE, FI, and SI came out to be the most common and significant direct determinant factors that explain technology adoption behavior. The results also suggest that the use of many moderating and core determinant factors to artificially achieve high predictability, is unnecessary and good predictive power can be achieved even with simple models of the technology adoption model. For example, where technology use is not mandatory for the user, as in this case study on public participation in monitoring execution of PIPs in Nigeria [45], voluntariness is not an applicable moderating factor. Several authors [27,37,49] show that moderating factors can be context-dependent. Therefore, hedonic motivation, price value, and habit may not be unique to achieving higher predictability in an exploratory study of the public's behavioral intention to use e-government tools (BIE) to participate in monitoring execution of PIPs than can be observed from only the four core determinants [50,51]. Similarly [52], adapted the UTAUT model to adoption of electronic document management system (EDMS) and concluded that PE, EE, and SI are adequate, direct determinants of behavioral intention to use EDMS technology without any moderating factors. Ibrahim et al. [51], also proposed a similar UTAUT model with the four core determinants as direct determinants of behavioral intention to use e-government services, which also was similar to the adapted UTAUT model for google chrome cast adoption in Taiwan [53].

Maznorbalia and Awalluddin [27] consolidated these reviews by adapting a four-core-determinant version of the UTAUT model to study user acceptance of e-government in Malaysia, which presented “a complete picture of the acceptance process than any previous individual models.” Thus, a simple UTAUT model with the four core direct determinants of behavioral intention to use e-government tools (BIE) to monitor PIPs execution could be used for exploratory studies on a government-public engagement phenomenon in the Nigerian context, which is a knowledge gap in current literature on e-government (Table 1).

### 2.3 Proposed e-government adoption model and hypotheses for public participation in monitoring PIPs execution in Nigeria

**Fig. 2:**
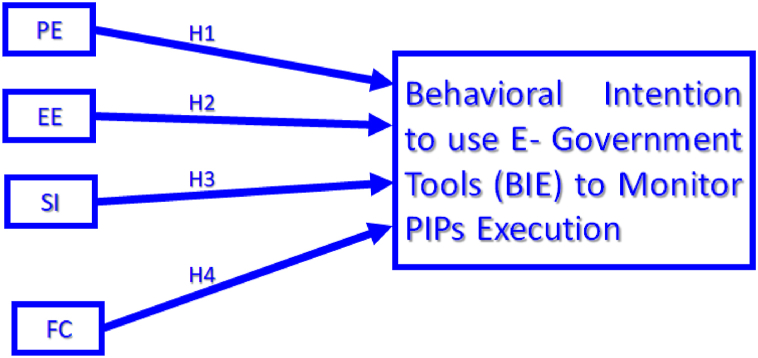
Proposed UTAUT model for adopting e-government tools for public participation in PIP monitoring; PE—performance expectancy, EE—effort expectancy, SI—social influence, FC—facilitating conditions, PIPs—public infrastructure project, BIE- Behavioral Intention to use E-government tools, UTAUT—Unified theory of acceptance and use of technology model.

The UTAUT model and its robust variations has been widely applied to technology use and adoption in several scenarios and contexts. Its reliability has been consistently validated, confirming its generalizability and supporting the argument that it serves as a benchmark for technology use and adoption studies [27,37,49,54,55]. Thus, this study proposes a simple model adapted from the UTAUT model. The model directly links each of the four constructs, the core determinants of behavioral intention to use e-government tools (BIE) for monitoring PIPs execution (Fig. 2 and Table 2), as independent variables to BIE, where BIE is the dependent variable. In addition, considering our literature survey and to the best of our knowledge, none of the previous studies were particularly extended to government-public engagement in management of PIPs in Nigeria (Table 1) which this proposed model (Fig. 2) addresses.

**Table 2:** Key constructs for the model of public behavioral intention to use e-government tools for monitoring execution of public infrastructure projects in Nigeria.

Construct	Description
Performance expectancy (PE)	The degree to which an individual believes using e-government tools and platforms will help attain a higher level of participation in monitoring PIPs execution; this expectancy regards saving time used for participation, enhancing effectiveness in participation by assessing the most relevant information on the project's progress, enhancing productivity via prompt engagement with government or project team to prevent project failure, enhancing convenience to participate in project monitoring and overall usefulness of the e-government system to the individual [27,56,57].
Effort expectancy (EE)	The degree to which an individual believes using e-government tools and platforms to monitor PIPs execution will be easy; this attribute regards learning how to operate and navigate the e-government tools and platforms, clarity in interaction with the e-government tools and platforms, ease of becoming skillful at using the system, and overall ease of using the system [27,44,51,57].
Social influence (SI)	The degree to which an individual considers how others think they should use e-government tools and platforms to monitor the execution of PIPs, to be important; the social influence regards how much the thoughts, opinions, and behavior of other people towards the use of e-government tools and platforms to monitor PIPs execution influence an individual's intention to use e-government tools and platforms to monitor PIPs execution [27,44,45,51,52].
Facilitating conditions (FC)	The degree to which an individual believes organizational and technical infrastructure exists to support the use of e-government tools and platforms to participate in monitoring PIPs execution; the conditions include having resources, knowledge, enough previous Internet use or mobile Internet use experience, a help structure such as help desk or network of people to help individuals facing challenges to use e-government tools and platforms to monitor PIPs execution [36,44,45,51,52].
Behavioral intention	The degree to which an individual intends to use e-government tools and platforms to monitor PIPs execution. The measure expresses the strength of intention to use e-government tools and platforms to monitor PIPs execution [44,45].

Judging from e-government indices, Nigeria is currently at a low level of readiness and user-experience with e-government adoption and public participation in monitoring PIPs execution [9,58]. This study will close the gap in literature on public willingness to adopt e-government tools to participate in monitoring PIPs execution in Nigeria. As a result, it will contribute towards reduction in number of failed or abandoned PIPs in Nigeria.

From a public user perspective, as per the modified UTAUT model, BI to use e-government approaches to monitor PIP execution is directly influenced by public perception on PE, EE, FC, and level of SI associated with the use of e-government tools and platforms.

From the literature review, the study proposes the following hypotheses (see Fig. 2).

H1 Public perception of PE from the use of e-government tools to monitor PIPs execution has a significant positive impact on public's behavioral intention to use e-government tools (BIE) to monitor PIPs execution.

H2 Public perception of EE associated with the use of e-government tools to monitor PIPs execution has a significant positive impact on public's BIE to monitor PIPs execution.

H3 Public perception of the SI level associated with the use of e-government tools to monitor PIPs execution has a significant positive impact on public's BIE to monitor PIPs execution.

H4 Public perception of FC for the use of e-government tools to monitor PIPs execution has a significant positive impact on public's BIE to monitor PIPs execution (BIE).

## 3 Methodology

This study employes a quantitative research method (Fig. 2 and Table 2) using a questionnaire to measure the adapted UTAUT constructs. The questions in the questionnaire were innovatively adapted by the authors from previous studies on e-government [44,45,56] to suit the Nigerian context. For the data analysis, this study adopts a sequential exploratory factor analysis (EFA) and structural equation modelling (SEM) - confirmatory (CFA) factor analysis approach to identify underlying constructs and determine measurement model fit for a path model of the constructs. It further uses SEM-path analysis to test the hypotheses on extent of relationship between the variables. IBM Statistical Package of the Social Sciences (SPSS) version 28 and Analysis of Moment Structure (AMOS) version 26 software were used for statistical analysis of the data. Separate survey data and results were used for the EFA and CFA. This approach is suited for exploratory research that seeks to understand a phenomenon and does not involve experimental settings for testing a cause-effect relationship using a “treatment group” [59,60].

### 3.1 Survey questionnaire instrument: face and content validity

This study developed a questionnaire measurement instrument (Appendix A) to gather data. It comprises two sections. Section 1 contains background information questions, general questions about the technologies respondents use to access the Internet, and questions on their involvement in resolving government issues and problems. Section 2 contains questions to measure respondents’ perception of the behavioral factors influencing public willingness to adopt e-government tools to participate in monitoring PIPs execution. The questions in Section 2 were developed from questions validated and used in previous UTAUT studies and adapted to the public project management and Nigerian contexts. This study employed a five-point Likert scale for the questions (1 = Strongly disagree, 2 = Disagree, 3 = Neither Agree Nor Disagree, 4 = Agree, and 5 = Strongly Agree). The original survey instrument by Ref. [44] has been adapted and validated to capture the behavioral factors for technology acceptance in several studies and contexts [27,[50], [51], [52]].

Before distributing the survey, the study conducted a pilot test for face and content validity of survey questions. The questionnaire was first sent to 14 professionals (seven in the US and seven in Nigeria), who are either experts or familiar with project management, to validate the questions. The fourteen professionals were purposely selected from project management and academic research professional networks to provide feedback to the authors based on their experiences with either project management or academic researches. It was then sent to the Institutional Review Board (IRB) at the University of Alaska Fairbanks for review. After implementation of comments from the pilot test and reviews, the questionnaire was administered to two separate population samples to get respective results for the EFA and CFA. Where EFA and CFA are to be conducted for the same study, a different population sample and survey result data should be used for the two analyses [[61], [62], [63]].

### 3.2 Population sample

The population for the survey is the general public of Nigeria that has attained the age of 18 years or above (suffrage age in Nigeria), which is a population of approximately 120 million [64]. For the EFA that merely test for underlying factors, the entire country was taken as the unit of analysis. For the CFA that test for extent of relationship between the variables, the six geopolitical regions were taken as the unit of analysis. CFA requires a large and widely spread sample size. The hard copy questionnaire was randomly administered at public transport terminals and markets. Online questionnaire survey developed on SurveyMonkey was also randomly distributed by email, text, and WhatsApp platforms between April through December 2022.

## 4 Results and test of hypotheses

### 4.1 Exploratory factor analysis (EFA)

The EFA used principal component analysis and Promax rotation in the SPSS version 28 software to identify and validate underlying common factors and latent constructs. Though the factors have been identified from the forerunner UTAUT studies, the data set of results from the EFA survey helped validate the questionnaire items (observed variables) loading to their respective constructs (latent variables) and demonstrate the questionnaire's fit for the subsequent CFA in the Nigeria and project management contexts.

#### 4.1.1 Sample size results for EFA

The in-person and online survey yielded 498 questionnaires, of which 26 were rejected for incomplete responses, leaving 472 completed questionnaires for data analysis, at a ratio of 79% from online respondents to 21% from in-person respondents. For large population surveys [65], a minimum randomly selected sample size of 385 respondents for a unit of analysis is adequate to achieve a 95% confidence level and a 5% margin of error.

#### 4.1.2 EFA results

A minimum individual factor loading (β) threshold of 0.6 for construct validity, minimum Cronbach alpha (α) of 0.7 for scale reliability of each construct, and Kaiser-Mayer-Olkin (KMO) measure of sampling adequacy score between 0.8 and 1 for model fit to the data, were adopted for the EFA [66].

**Fig. 3:**
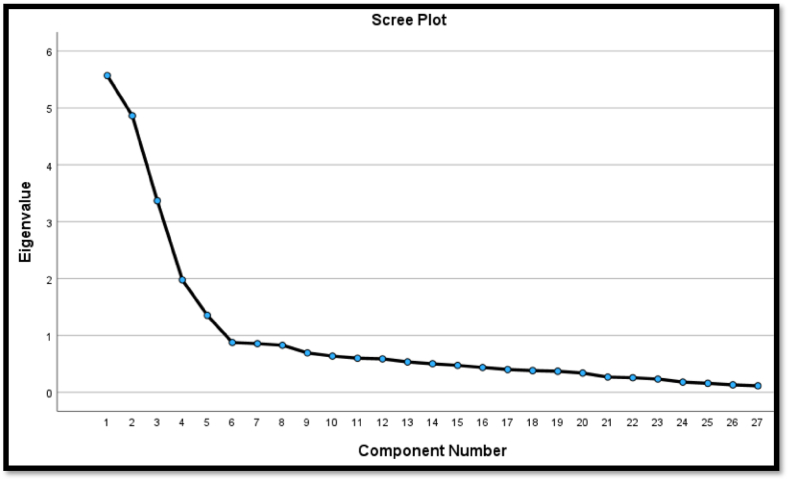
Scree plot from EFA

The EFA results using SPSS (Table 3) shows that each questionnaire item (observed variable) loaded into its appropriate construct (latent variable) in the questionnaire, exceeding the minimum thresholds, except for RBIE7. Thus, RBIE7 was disregarded in the conceptual measurement model for the CFA analysis. It means RBI7 does not contribute significantly to the convergent validity and measurement of BIE. The EFA results identified components 1(BIE), 2(PE), 3(FC), 4(SI), and 5 (EE). The scree plot result (Fig. 3.) also identified five underlying components in the result dataset based on the elbow position with a cumulative total variance explained of 63.4% based on the eigenvalues from SPSS. The KMO measure of sampling adequacy was 0.83 demonstrating a good model fit to the data set. These results validate reliability of the scale of measurement used in the questionnaire and align with prior studies on technology adoption [27,50]. The component factors—BIE, PE, SI, FC, and EE—are distinct constructs and underlying factors for e-government adoption to monitor PIPs execution. A subsequent CFA will further validate the extent of relationship between the constructs.

**Table 3:** Exploratory factor analysis results: Factor loading from EFA survey data. Pattern Matrixa.

Questionnaire item	Mean	Component					Cronbach	Eigenvalues	
		1	2	3	4	5			
Alpha	% of Variance	Cumulative % of Variance							
BIE1	3.97	0.726					0.89	20.62	20.62
BIE2	4.09	0.862							
BIE3	4.02	0.741							
BIE4	4.05	0.874							
BIE5	4.05	0.808							
BIE6	4.27	0.802							
RBE7	4.08						Disregarded		
PE1	4.29		0.777				0.88	18.61	38.64
PE2	4.24		0.849						
PE3	4.30		0.810						
PE4	4.33		0.773						
PE5	4.21		0.770						
FC1	4.11			0.905			0.91	12.47	51.11
FC2	4.12			0.908					
FC3	4.12			0.824					
FC4	4.13			0.665					
FC5	4.10			0.918					
SI1	4.00				0.677		0.75	7.32	58.43
SI2	4.10				0.737				
SI3	4.11				0.822				
SI4	4.15				0.614				
SI5	4.12				0.711				
EE1	4.14					0.822	0.88	5.01	63.34
EE2	4.16					0.824			
EE3	4.19					0.644			
EE4	4.19					0.710			
EE5	4.21					0.631			

Extraction Method: Principal Component Analysis; Rotation Method: Promax with Kaiser Normalization.

a. Rotation converged in six iterations.

### 4.2 Confirmatory factor analysis (CFA)

This study employed CFA to test the measurement model of the constructs identified by the EFA (i.e., BIE, PE, SI, FC, and EE) and the fit for a subsequent path model using SEM techniques. A separate survey with the same validated questionnaire used for the EFA, was used to gather a separate result dataset for the CFA. The collected data was analyzed using SPSS AMOS. SPSS was used for basic descriptive statistics and SPSS AMOS was used to test the validity and model fit of the measurement model for the constructs. The selected measurement model was subsequently modified into a path model to test the hypotheses and determine the extent of relationships between the constructs.

#### 4.2.1 CFA: sample size results

The in-person and online survey yielded 2758 questionnaires from respondents, of which 252 were rejected for incomplete responses, leaving 2506 completed questionnaires for data analysis. About, 65% of the respondents were from the online survey.

#### 4.2.2 CFA: demographic characteristics of respondents

Table 4 shows the main survey respondents across the states in Nigeria. Between the six major geographical regions in Nigeria, the relative percentage of the sample size is similar to the actual relative population proportion in Nigeria. Furthermore, each of the six geopolitical regions has a minimum of 300 respondents, which is enough to achieve 95% confidence level and 6% margin of error. There is an adequate representative spread of respondents across the six geopolitical regions in Nigeria. The gender balance of 49% female to 51% male is also moderately representative, making the result non-gender biased. The age distribution is also representative, with about 70% of respondents between the age of 18 years and 39 years, which reflects the younger age demographic of the Nigerian adult population, most of whom had about six to nine years of school education (Table 5). Out of the total number of respondents, 75% have computers and 99% have mobile phones. Agreement with statement ‘I expect to use mobile phone more than computer to access internet in the future” has an average score of 4.3 in a scale of 5.0.

**Table 4:** Respondents by regions in Nigeria.

No.	Geopolitical region	Number of respondents (Percentage of sample size)		Percentage of Nigerian population
1	North-West (NW)	621 (25%)		27%
2	North-East (NE)	356 (14%)		11%
3	North-Central (NC)	396 (16%)		15%
4	South-West (SW)	479 (19%)		19%
5	South-East (SE)	303 (12%)		10%
6	South-South (SS)	351 (14%)		17%

**Table 5:** Demographic profiles of samples (N = 2506).

Demographic profile	Category	Frequency	Percentage (%)
Gender	Male Female	1265 1241	51 49
Age	18–19 years 20–29 years 30–39 years 40–49 years 50+ years	601 599 563 467 276	24 24 22 19 11
Highest level of Education	University degree or equivalent Secondary School Certificate Others	1453 740 313	58 29 13

#### 4.2.3 CFA: descriptive statistics results

The mean score for each questionnaire item and all items under the same construct (Table 6) is greater than or almost reaching 4.0. Respective average BIE score for NW, NE, NC and SW regions is 4.1, while for SE and SS, it is 4.0. At face value, the results reflect a strong positive opinion on the importance of the predictor factors and intention to adopt e-government tools to participate in monitoring PIPs execution in Nigeria. For each questionnaire item, the Skewness is <|3| and Kurtosis is <|10|, demonstrating the normality of each variable of the constructs and supporting the use of maximum likelihood estimates to analyse the relationships between the variables [67,68].

**Table 6:** Descriptive statistics and construct validity results.

Construct	Item	β_1_	β_2_	Mean	SD	Skp	Κ	α	Mean	SD	AVE	CR
Effort Expectancy (EE)	EE1	0.79	0.80	4.13	0.67	−1.38	4.59	0.85	4.14	0.52	0.53	0.85
	EE2	0.73	0.73	4.13	0.63	−1.40	5.57					
	EE3	0.78	0.78	4.10	0.70	−1.58	5.42					
	EE4	0.64	0.64	4.20	0.65	−1.02	2.91					
	EE5	0.69	0.69	4.14	0.63	−1.48	6.30					
Performance Expectancy (PE)	PE1	0.71	0.71	4.29	0.71	−1.73	6.22	0.86	4.27	0.54	0.56	0.86
	PE2	0.81	0.82	4.27	0.66	−1.33	4.70					
	PE3	0.76	0.76	4.27	0.67	−1.32	4.24					
	PE4	0.82	0.82	4.31	0.71	−1.48	4.50					
	PE5	0.63	0.63	4.21	0.62	−1.13	4.76					
Facilitating Condition (FC)	FC1	0.61	0.61	4.14	0.60	−1.50	7.54	0.73	4.16	0.50	**0.41**	0.73
	FC2	0.68	0.70	4.12	0.62	−1.65	7.65					
	FC3	0.64	0.64	4.22	0.68	−1.54	5.76					
	FC4	0.62	0.61	4.16	0.78	−1.59	4.33					
	**FC5**	**0.51**	Deleted									
Social Influence (SI)	SI1	0.79	0.80	3.96	0.79	−1.76	4.49	0.87	4.02	0.66	0.63	0.87
	SI2	0.86	0.87	4.01	0.75	−1.72	4.90					
	SI3	0.80	0.79	4.02	0.76	−1.69	4.73					
	**SI4**	**0.51**	Deleted									
	SI5	0.71	0.70	4.08	0.82	−1.64	4.02					
e-Government (BIE)	BIE1	0.73	0.73	3.94	0.67	−1.89	6.46	0.87	4.05	0.55	0.54	0.87
	BIE2	0.81	0.81	3.98	0.67	−1.75	6.33					
	BIE3	0.77	0.77	4.06	0.72	−1.55	4.83					
	BIE4	0.77	0.77	3.98	0.70	−1.61	5.08					
	BIE5	0.73	0.73	3.98	0.68	−1.70	6.08					
	BIE6	0.59	0.59	4.36	0.78	−1.78	4.84					
	**RBIE7**	**0.28**	Deleted									

Note: Red items have individual factor loading significantly <0.6 and will be deleted to arrive at the selected measurement model; SD—Standard deviation, Skp—Skewness, K—Kurtosis, β_1_—Factor loading for conceptual measurement model, β_2_—Factor loading for selected measurement model, α—Cronbach alpha, AVE—Average variant extracted, CR—composite reliability.

#### 4.2.4 CFA: conceptual and selected measurement models -reliability, validity and model fit results

**Fig. 4:**
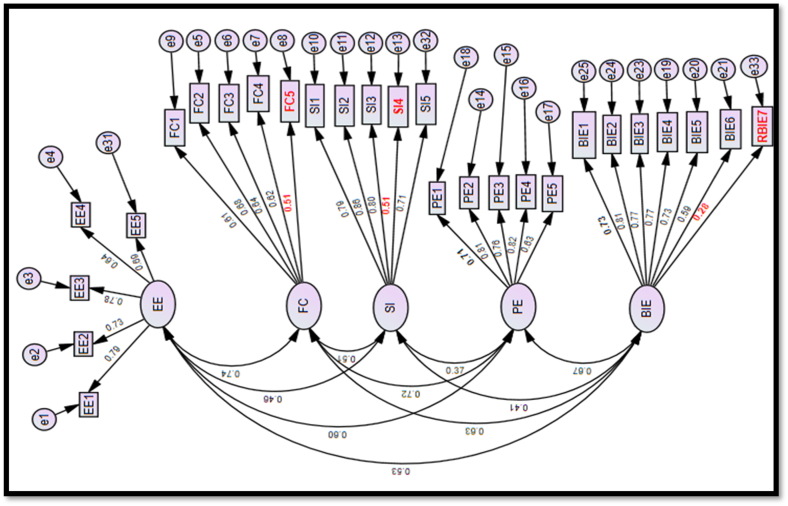
Conceptual measurement model includes the latent factors EE-effort efficiency, FC -facilitating condition, SI -social influence, PE-performance expectation, BIE - behavioral intention to use e-government and all their respective questionnaire items from the EFA.

The study developed a conceptual measurement model for the constructs (Fig. 4), based on results from the EFA. Using the CFA result dataset, the model was tested with SEM for individual reliability and model fit to be a selected measurement model for subsequent path analysis.

**Fig. 5:**
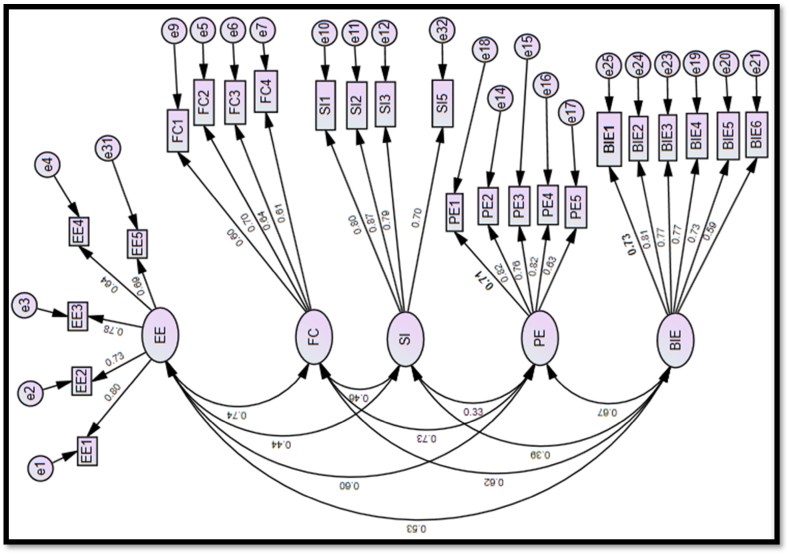
Selected Measurement Model includes the latent factors EE-effort efficiency, FC -facilitating condition, SI -social influence, PE-performance expectation, BIE - behavioral intention to use e-government and only their respective questionnaire items with factor loading greater than 0.6.

Construct validity for each question was evaluated by comparing the individual β_1_ of each item against a minimum threshold of 0.6 [66]. All questionnaire items (Table 6) met this requirement except for items FC5 (0.51), SI4 (0.51), and RBIE7 (0.28), which would be deleted to arrive at the selected measurement model for the path analysis. The β_1_ for BIE6 (0.59) is almost 0.6; thus, it was retained in the selected measurement model (Fig. 5).

The selected measurement model (Fig. 5) was determined by deleting items with β_1_ significantly <0.6 (FC5, SI4, and RBIE7) from the conceptual measurement model. Reliability and validity tests were then conducted on the selected measurement model. From the construct validity tests, all remaining questionnaire items in the selected measurement model met the minimum threshold (individual β_2_ score of 0.6) in Table 6, except for item BIE6 with β_2_ of 0.59. Given that it was almost 0.6, the study retained it in the selected measurement model. For the reliability tests, all constructs have α > 0.7. For convergent validity, all constructs have β_2_ > 0.6, CR score ≥0.6, and AVE of ≥0.5 respectively, except FC with AVE of 0.41. However, with a complementary CR ≥ 0.6, it meets the convergent validity requirements [56]. Discriminant validity was tested with the Heterotrait-Monotrait (HTMT) ratio discriminant validity test method. From Table 7, the selected measurement model satisfies the discriminant validity threshold of HTMT ratio of less than 0.9 for each of the items.

**Table 7:** Discriminant validity results— heterotrait-monotrait ratio method.

	PE	EE	FC	SI	BIE
**PE**					
**EE**	0.63				
**FC**	0.74	0.74			
**SI**	0.35	0.45	0.46		
**BIE**	0.70	0.55	0.62	0.39	

Note: PE—performance expectancy, EE—effort expectancy, SI—social influence, FC—facilitating conditions, BIE—Behavioral intention to adopt e-government tools to monitor PIPs execution.

#### 4.2.5 CFA: Model's goodness of fit results

Table 8 presents the goodness of fit (GoF) test results for the selected measurement model to demonstrate its fit for the subsequent path model or structural component analysis. The model met all GoF requirements either at excellent or moderate fitness and validated the model fit for path or structural component model. The Chi-square/df value is 15.3, expected for large sample sizes [[69], [70], [71]].

**Table 8:** Goodness of fit results and criteria for selected measurement model.

Criteria	Score	Comment	Criteria Excellent Fit	Criteria Moderate Fit	Reference
SRMR	0.05	Acceptable fit	<0.05	<0.08	[71,72]
RMSEA	0.076	Acceptable fit	<0.05	<0.08	[70,72]
GFI	0.88	Acceptable fit	>/ = 0.9	>/ = 0.8	[70,72]
AGFI	0.85	Acceptable fit	>/ = 0.9	>/ = 0.8	[70,73]
NFI	0.88	Acceptable fit	>/ = 0.9	>/ = 0.6	[70]
RFI	0.87	Acceptable fit	>/ = 0.9	>0.8	[74,75]
IFI	0.89	Acceptable fit	>/ = 0.85	>/ = 0.8	[76]
CFI	0.89	Acceptable fit	>/ = 0.9	>/ = 0.6	[70,72]
*P*-value	0.00	Acceptable fit			

Note: SRMR-standardised root mean square residual, RMSEA -root mean square error of approximation, GFI-goodness of fit index, AGFI-aggressive goodness of fit index, NFI-normed fit index, RFI-relative fit index, IFI-incremental fit index, CFI-comparative fit index.

Overall, the selected measurement model for the public's adoption of e-government to participate in monitoring PIPs execution has an acceptable fit, demonstrating convergent and discriminant validity. Hence, the selected measurement model is a good fit for a path model analysis using SEM techniques to test the hypotheses.

### 4.3 Path model and hypotheses testing

**Fig. 6:**
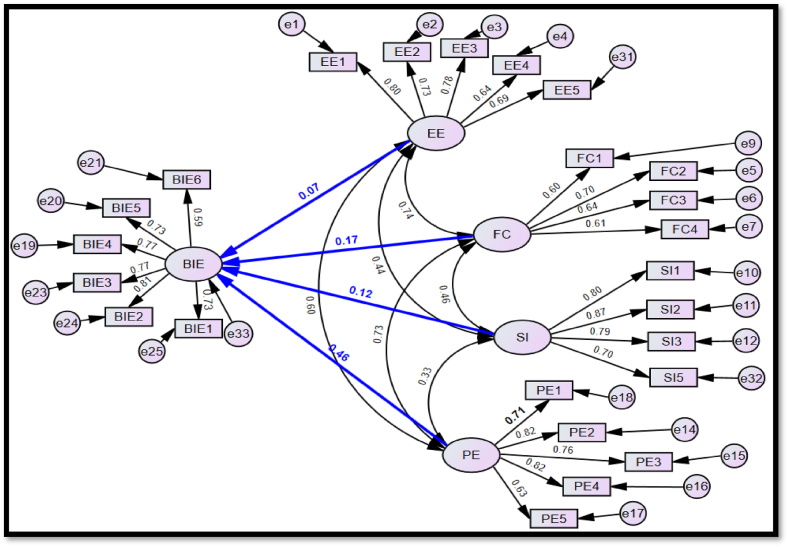
Relationship path model includes all latent factors EE-effort efficiency, FC -facilitating condition, SI -social influence, PE-performance expectation, BIE - behavioral intention to use e-government, their directional relationship arrows, and their respective questionnaire items from the selected measurement model.

A relationship path model (Fig. 6) to represent H1, H2, H3, and H4 was developed in SPSS AMOS for the variables in the selected measurement model (Fig. 5). The selected measurement model was modified into a path model to test the hypotheses and determine extent of relationships between the constructs. Using SPSS AMOS and applying SEM techniques for path analysis, the hypotheses were tested by determining the standardised path coefficient for relationship paths between PE and BIE (H1), EE and BIE (H2), SI and BIE (H3), and FC and BIE (H4) and validating if the relationships are statistically significant at a *P*-value of 0.05 or 95% level of significance. The path model (Fig. 6) meets all GoF requirements (Table 9) at excellent fit level, supporting its fit for testing the hypotheses. SEM is based on a covariance matrix to analyse the relationship between variables and is good for testing hypotheses [77,78].

**Table 9:** Goodness of fit results and criteria for the path model.

Criteria	Score	Comment	Criteria -Excellent Fit	Criteria -Moderate Fit
SRMR	0.05	Acceptable fit	<0.05	<0.08
RMSEA	0.076	Acceptable fit	<0.05	<0.08
GFI	0.88	Acceptable fit	>/ = 0.9	>/ = 0.8
AGFI	0.85	Acceptable fit	>/ = 0.9	>/ = 0.8
NFI	0.88	Acceptable fit	>/ = 0.9	>/ = 0.6
RFI	0.87	Acceptable fit	>/ = 0.9	>/ = 0.8
IFI	0.89	Acceptable fit	>/ = 0.85	>/ = 0.8
CFI	0.89	Acceptable fit	>/ = 0.9	>/ = 0.6
P	0.00	Acceptable fit		

Note: SRMR-standardised root mean square residual, RMSEA -root mean square error of approximation, GFI-goodness of fit index, AGFI-aggressive goodness of fit index, NFI-normed fit index, RFI-relative fit index, IFI-incremental fit index, CFI-comparative fit index.

#### 4.3.1 Path model and hypotheses testing results

Table 10 presents the SEM path analysis results for the model. The path relationship between PE and BIE, EE and BIE, SI and BIE, and FC and BIE have a standardised factor or path coefficient of 0.46, 0.07, 0.12, and 0.17, respectively (Fig. 6, Table 10). Based on the SEM results, H1, H2, H3, and H4 are supported at the 95% level of significance. Moreover, PE (β = 0.46; P value = 0.000), EE (β = 0.07; P value = 0.03), SI (β = 0.12; P value = 0.000), and FC (β = 0.17; P value = 0.000) show positive effects on the public's behavioral intention to use e-government tools (BIE) to participate in monitoring PIPs execution.

**Table 10:** Hypotheses testing results.

Hypothesis (Factor)	Path	Standardised Beta (Unstandardised Beta)	SE	CR	P	Comment
H1 (PE)	PE →BIE	0.46 (0.46)	0.03	14.18	***	Accept
H2 (EE)	EE →BIE	0.07 (0.07)	0.03	2.16	0.03	Accept
H3 (SI)	SI →BIE	0.12 (0.10)	0.02	5.85	***	Accept
H4 (FC)	FC →BIE	0.17 (0.21)	0.06	3.88	***	Accept

R^2^ squared multiple correlation is 0.50.

Note: *** less than 0.001; PE—performance expectancy, EE—effort expectancy, SI—social influence, FC—facilitating conditions, BIE—behavioral intention to adopt e-government tools to monitor PIP execution, CR—composite reliability, SE-scalar estimate, *P*-level of significance.

## 5 Discussion, conclusions and recommendations

### 5.1 Discussion of findings

Based on the results from the CFA, Fig. 2 is established as the UTAUT framework for identifying factors that facilitate the public to adopt e-government tools for monitoring PIPs execution in Nigeria context. The model and results for this research indicates a strong behavioral intention to adopt e-government tools and techniques (BIE) to monitor PIPs execution amongst the Nigerian public. The overall average response score for public's BIE to monitor PIPs execution in Nigeria is 4.05 on the five-point Likert scale. The Nigerian public is willing to participate in the monitoring of PIPs execution, which seems counterintuitive, as several studies empirically observe low public participation levels in other public issues, such as participatory budgeting, land use, and natural resource management, in Nigeria [[79], [80], [81]]. Thus, e-government adoption may signal hope for the currently observed low level of public participation in other public issues in Nigeria, as also noted by Refs. [14,82].

From the EFA, four determinant factors (PE, FC, SI, and EE) that influence public's BIE to monitor PIPs execution were identified and validated. The results agree with findings from prior UTAUT studies on technology adoption [60,61]. From the SEM path model analysis of the variables and empirical data from the Nigerian public, the perception of PE, FC, SI, and EE were found to positively influence and collectively explain 50% of variations (R^2^ = 0.50) of the data on public's BIE to monitor PIP execution. This result aligns with previous studies on application of UTAUT model to predict behavioral intention to adopt e-government to participatory budgeting in Portugal and behavioral intention to use internet voting in local election in Slovenia [10,12]. Similarly, a modified UTAUT model applied to adoption of solar water pump for agriculture in Nigeria was able to explain about 60% of variance in the data even though the model had an additional determinant factor, “awareness level” besides PE, EE, FC, and SI [83].

Among the four factors, PE has the highest positive support for public's BIE to monitor PIPs execution in Nigeria. This result aligns with prior studies on the adoption of ICT in other disciplines, such as mobile nursing [84], mobile payment for shopping [85], and mobile learning in postgraduate studies [86], where PE likewise has the highest positive support for behavioral intention to adopt ICT tools. The Nigerian public believes that if the adoption of e-government tools can help save time to access, efficiently share, and effectively discuss information and participate in decision-making for PIPs, they will be willing to adopt e-government tools to monitor execution of the projects. Similar results were obtained in application of UTAUT to internet voting in Portugal and public adoption of technology to management of COVID in Nigeria [12,14].

The results also show that FC and SI have positive influence on the public's BIE to monitor PIPs execution in Nigeria, similar to results obtained by Refs. [11,14,19]. It means that if people with social influence can take visible lead to post comments on progress of on-going PIPs on the project websites, more people from the public will be influenced to also engage with the projects via the websites. Another implication from the results is that the Nigerian public will like to see some facilitating conditions in place to incentivize them to effectively adopt e-government tools to monitor PIPs execution. Interestingly, the questionnaire items with the highest average score for FC are FC3 (importance of getting basic facilities like electricity, bandwidth etc., to access the internet) and FC4 (importance of having basic needs of food, health and security at least met at the bare minimum first before effectively using e-government platforms to participating in monitoring of the project). This coincides with results obtained from application of modified UTAUT model to behavioral intention to adopt technology to manage COVID in Nigeria [14]. As noted by Ref. [14], reliable and affordable high speed internet services and constant electricity supply may help incentivize the public to effectively adopt e-government tools and practices to participate in public issues such as monitoring PIPs execution in Nigeria.

From the results, EE has the least positive support for public's BIE to monitor PIPs execution in Nigeria. This observation aligns with [84], where EE does not support the behavioral intention to use a mobile nursing app by nurses in a Chinese hospital. According to Ref. [87], perceived ease of use or concern about the effort to be proficient at using a technology tool is important only at the early stage of its introduction or adoption. Hence if a technology tool has already transitioned from the early-adoption to post-adoption phase, such as the use of mobile phones to access the Internet, which is prevalent in Nigeria, EE may not strongly influence the behavioral intention to adopt the technology for other applicable services. This situation may explain why EE has the least positive support for public's BIE to monitor PIPs execution in Nigeria. Mobile phones with internet capabilities are so widely used in Nigeria that the learning curve on its adoption to monitor project information should not be steep.

Overall, from the public perspective, PE, FC, SI, and EE positively influence public's BIE to monitor PIPs execution in Nigeria, which answer the research question. Similar to previous related studies, these four factors form the base direct determinant factors for the public's BIE to monitor PIPs execution in Nigeria.

### 5.2 Conclusions and recommendations

E-government has been presented as a good enabler to public participation. This study answered the research question on factors perceived by the Nigerian public to influence their BIE to monitor PIPs execution. This study successfully modified the UTAUT model and questionnaire instruments from previous studies on the adoption of e-government or technology to develop a conceptual model that suits the discipline of public project management and context of public participation in monitoring PIPs execution in Nigeria. The results support adaptation of the UTAUT model as a base model that policymakers, project managers, and scholars can adopt to make new policies, evaluate and predict the public's BIE to monitor PIPs execution in Nigeria.

#### 5.2.1 Contributions to practice and policy implications

From the public perspective, PE, FC, SI, and EE are found to positively influence the BIE to monitor PIPs execution in Nigeria. Based on the findings, the Nigerian government and civil society must find ways to enhance fulfilment of the determinant factors such as mandating each PIP to have an active, up-to-date, and efficient website and app to publicly share progress information, leveraging social media to encourage more public participation, and implementing plans and policies that will wipe away the digital divide and open wider access to the internet and mobile telecommunication services. Every effort to meet the determinant factors’ requirements will drive the public to a stronger level of BIE to monitor PIPs execution in Nigeria, which could subsequently translate to the actual adoption of e-government tools and contribute towards sustainable and better infrastructure development, and quality of life.

Accordingly, the government should develop policies that (i) require project teams to set up e-government platforms (e.g., websites and mobile apps) to provide progress information on PIPs that can be efficiently and easily accessed by the public. (ii) intentionally encourage active and efficient engagement between the government officials, PIP teams, and the public on issues related to PIPs via the project's e-government platforms. If the public perceived the e-government platform to be moribund and inefficient, they will not continue with its adoption to participate in monitoring projects. (iii) encourage PIP websites to be actively linked to social media platforms to create social influence and awareness to keep more people engaged with the platforms. Social media networks and peer influence can be leveraged to motivate the public to adopt e-government tools to monitor PIP execution in Nigeria. The capacity to link project websites to other social media sites could provide some good measure of SI that will positively influence BIE to monitor PIPs execution in Nigeria and (iv) incentivize stakeholders in the telecommunications sector to actively work towards bridging the digital divide from the standpoint of accessibility, affordability, and availability of e-government supporting infrastructure to the public. For example, government can give permitting waivers to telecommunication companies that are installing broadband internet facilities in rural areas.

#### 5.2.2 Contributions to research

Despite several studies on the adoption of e-government tools and practices in different fields, few examine the application of e-government tools and practices to government-public engagement in project management particularly, public participation to monitor PIPs execution in Nigeria (Table 1). Based on the literature review, this study is among the first to apply the UTAUT model to the adoption of e-government tools and practices for public participation in monitoring PIPs execution in Nigeria. This study developed a conceptual model that extends the frontier of e-government to the discipline of project management especially PIPs in Nigeria and provides another validation data for UTAUT model. It contributes to closure of gap in literature on application of UTAUT model to public infrastructure project management and lack of empirical studies on this topic. It provides empirical validation of a base model of UTAUT to evaluate and predict behavioral intention to adopt e-government in government-public engagement to monitor PIP execution in Nigeria.

### 5.3 Limitations

Although this study offers important theoretical and practical implications, it has a few limitations and further research should be done. First, this study was limited to finding out the behavioral intention of the public to use e-government tools to participate in monitoring PIPs execution in Nigeria. Currently, Nigeria has little or no actual use experience with adoption of e-government tools to monitor PIPs execution. Although previous findings have reported that intention to act is positively correlated with actual use behavior [88], future studies should investigate relationship between the public's behavioral intention to use and actual use behavior of e-government to monitor PIP execution, as Nigeria matures in adoption of e-government in the near future [9].

Second, the context of this study was the Nigerian public. Despite being similar to other sub-Saharan African countries, Nigeria may be peculiar in specific ways. This study may need to be adapted before it is generalized to other countries and situations.

## Author contribution statement

Peace Afieroho: Conceived and designed the experiments; Performed the experiments; Analyzed and interpreted the data; Contributed materials, analysis tools or data; Wrote the paper.

Xiyu (Thomas) Zhou: Robert Perkins: Bogdan Hoanca: Greg Protasel: Contributed materials, analysis tools or data; Wrote the paper.

## Data availability statement

Data will be made available on request.

## Additional information

No additional information is available for this paper.

## Declaration of competing interest

The authors declare that they have no known competing financial interests or personal relationships that could have appeared to influence the work reported in this paper.

## Appendix A Questionnaire

Section 1: Demographic and General Questions.

a. What is your age?
b. What is your gender?
c. What is the highest level of education you have completed?
d. Which State in Nigeria do you live?
e. Do you have a computer or a laptop?
f. Do you have a mobile phone?
g. To what extent do you agree with the following statements? ***Strongly Disagree, Disagree, Undecided, Agree, Strongly Agree***
  i. I expect to use mobile phone more than computer to access internet in the future.
  ii. I like to leave public issues and problems for government officials to handle.
  iii. I like to be involved in resolution of public issues and problems.

Section 2: Extent of agreement with the following statements.

Strongly Disagree, Disagree, Undecided, Agree, Strongly Agree.

a) What is your opinion on the following statements on perception of use of internet via computer or cellphone to access information, exchange information with government/project officials and engage with government/project officials in decision-making in monitoring execution of public infrastructure projects in your area?
  i. I plan to frequently go to the project website for timely project updates in the future. (BIE1)
  ii. I intend to regularly use comment and discussion features on the project website in the future. (BIE2)
  iii. I can see myself using the project website to engage with project officials and community members more than using in-person interaction. (BIE3)
  iv. I will likely post video clips and photos of project status on the project website in the future. (BIE4)
  v. I will likely attend a project meeting online via the project website in the future. (BIE5)
  vi. Overall, using the project website to engage with project officials and community members on the projects, is a good idea. (BIE6)
  vii. In the future, I will use comment and discussion features on the project website at least once a day; once a week; once week a month; once a year; and never. (BIE7) ****Recoded to a reverse scale as RBIE7***
b) I think using a project website to access information, exchange information with government/project officials and engage with government/project officials in decision-making in monitoring execution of public infrastructure projects in my area will require that
  i. I have the necessary knowledge or experience to use the website. (FC1)
  ii. I can get help when I have difficulties using the website. (FC2)
  iii. I can get basic facilities like electricity, bandwidth, etc to use the website. (FC3)
  iv. I have my basic needs of food, health and security at least met at the bare minimum first. (FC4)
  v. Overall, I have all it takes to use the website. (FC5)
c) I think using project website to access information, exchange information with government/project officials and engage with government/project officials in decision-making in monitoring execution of public infrastructure projects in my area will
  i. Enable me to participate more quickly. (PE1)
  ii. Enable me get access to relevant information and decision issues on public infrastructure projects in my area, more effectively. (PE2)
  iii. Enable me to participate more productively to prevent failure or abandonment of public infrastructure projects in my area. (PE3)
  iv. Enable me to participate more conveniently than participating face-to-face in monitoring execution of public infrastructure projects in my area. (PE4)
  v. Overall, be useful to me. (PE5)
d) I think making use of features on a project website to access information, exchange information with government/project officials and engage with government/project officials in decision-making in monitoring execution of public infrastructure projects in my area will
  i. Be easy to use and understand. (EE1)
  ii. Be easy for me to learn. (EE2)
  iii. Be easy for me to become skillful at. (EE3)
  iv. Not take much of my time and effort from my other daily activities. (EE4)
  v. Overall, be easy to use. (EE5)
e) Using a project website to access information, exchange information with government/project officials and engage with government/project officials in decision-making) in monitoring execution of public infrastructure projects in my area is something I would consider if
  i. People who are important to me think I should use it. (SI1)
  ii. People whose opinions I value would prefer me to use it. (SI2)
  iii. People who I value in society or my network influence me to use it. (SI3)
  iv. It is generally “cool” & trendy to use it. (SI4)
  v. Overall, other people important to me are using it. (S15)
